# Development of loop-mediated isothermal amplification (LAMP) for simple detection of *Leishmania* infection

**DOI:** 10.1186/s13071-015-1202-x

**Published:** 2015-11-14

**Authors:** Chaichontat Sriworarat, Atchara Phumee, Mathirut Mungthin, Saovanee Leelayoova, Padet Siriyasatien

**Affiliations:** Bangkok Christian College, Bangkok, 10500 Thailand; Department of Parasitology, Faculty of Medicine, Chulalongkorn University, Bangkok, 10330 Thailand; Department of Parasitology, Phramongkutklao College of Medicine, Bangkok, 10400 Thailand; Excellence Center for Emerging Infectious Diseases, King Chulalongkorn Memorial Hospital, Thai Red Cross Society, Bangkok, 10330 Thailand

**Keywords:** *Leishmania martiniquensis*, *L. siamensis*, LAMP, Malachite green, Diagnosis

## Abstract

**Background:**

Leishmaniasis is a neglected tropical disease that is caused by an obligate intracellular protozoan of the genus *Leishmania*. Recently, an increasing number of autochthonous leishmaniasis cases caused by *L. martiniquensis* and the novel species *L. siamensis* have been described in Thailand, rendering an accurate diagnosis of this disease critical. However, only a few laboratories are capable of diagnosing leishmaniasis in Thailand. To expand leishmaniasis diagnostic capabilities, we developed a simple colorimetric loop-mediated isothermal amplification (LAMP) technique for the direct detection of *Leishmania* DNA.

**Methods:**

LAMP was performed for 75 min using four primers targeting the conserved region of the18S ribosomal RNA gene, and the DNA indicator used was malachite green (MG). To simulate crude samples, cultured promastigotes of *L. siamensis* were mixed with blood or saliva. Also, clinical samples (blood, saliva, and tissue biopsies) were obtained from patients with cutaneous leishmaniasis (CL) and visceral leishmaniasis (VL). All samples were boiled for 10 min and introduced directly into the LAMP reaction mixture without DNA purification.

**Results:**

The use of MG resulted in an unambiguous differentiation of positive and negative controls. For *L. siamensis*, the detection limit was 10^3^ parasites/mL or 2.5 parasites/tube. Saliva, tissue biopsies, and whole blood were indicative of active *Leishmania* infection, and their direct usages did not adversely affect the detection limit. In addition, this LAMP assay could detect DNA from multiple *Leishmania* species other than *L. siamensis* and *L. martiniquensis*, including *L. aethiopica*, *L. braziliensis*, *L. donovani* and *L. tropica*.

**Conclusions:**

The simplicity and sensitivity of LAMP in detecting active *Leishmania* infection could enable the rapid diagnosis of leishmaniasis, thereby facilitating the survey and control of leishmaniasis in Thailand. However, our limited number of samples warranted a further validation with a larger cohort of patients before this assay could be deployed.

## Background

Causing more than 50,000 deaths annually [1], leishmaniasis is one of the most debilitating poverty-related diseases and presents a severe threat to socioeconomic development. This disease is caused by more than 20 species of the obligate intracellular protozoa *Leishmania* [2]. These are transmitted to humans through the bites of female sand flies [1]. Upon infection, three main clinical forms can be recognized: cutaneous leishmaniasis (CL), mucocutaneous leishmaniasis (MCL), and visceral leishmaniasis (VL). CL, the most common form, is characterized by the presence of various ulcerative lesions, which lead to disfiguring and/or disabling scars [3, 4]. Consequently, patients with CL often live in obscurity [5], thereby preventing expeditious treatment and increasing the probability of transmission. MCL is described by a severe destruction of mucosal regions (nose, mouth, and throat) [4]. VL is an infection of the internal organs that is characterized by prolonged fever, anemia, hepatosplenomegaly, and weight loss. VL is fatal if left untreated [6].

Since the first case report in 1996 [7], indigenous leishmaniasis has been increasingly prevalent in Thailand, especially in HIV patients. Case reports include both VL [7–12] and CL [10, 11, 13–15] and are concentrated in the northern and southern part of Thailand. In contrast to other regions in Asia, most cases in Thailand are caused by either *L. martiniquensis* or *L. siamensis* [12, 16]. As early detection is one of the most important aspects of disease containment, the need for a robust and rapid diagnostic method has never been higher. To summarize, currently available methods can be divided into three groups: parasitological methods, serological methods, and molecular methods, each of which presents various advantages and disadvantages.

Parasitological methods, which include microscopy and parasite culturing, have been considered the gold standard in diagnosing leishmaniasis. In Thailand, however, only a handful of laboratories could culture parasites. Also, serological diagnostic methods with comparable sensitivity to parasitological methods have been developed; however, most of them (enzyme-linked immunosorbent assay (ELISA) [17, 18], immunofluorescence antibody test (IFA) [19], and western blotting [20]) require sophisticated instruments, limiting their usages in healthcare environments. Moreover, these serological techniques have never been evaluated for the diagnosis of leishmaniasis in Thailand.

Molecular methods with great sensitivities and specificities have also been developed to diagnose leishmaniasis. One of the most classical techniques, polymerase chain reaction (PCR), is widely used [21–24]. However, the requirements for expensive equipment, DNA purification, and gel visualization have forestalled its utilization in field settings.

In 2000, Notomi et al. [25] developed the loop-mediated isothermal amplification (LAMP) method. In short, this method uses several complex primers and a strand-displacement polymerase to achieve amplification. Though conceptually challenging, LAMP has several advantages over PCR from a field diagnostics point of view. 1) The reaction proceeds isothermally, thereby obviating the need for expensive thermal cyclers [25]. 2) Crude DNA extracts can be used directly without purification [25, 26]. 3) The products can be detected visually using multiple parameters, including turbidity, fluorescence, and color.

Nevertheless, turbidity is challenging to discern and is unstable over time. Fluorescence measurement requires costly dyes (SYBR Green I [27], calcein [28], propidium iodide [29]) and is technically inconvenient due to its requirement for UV illumination. Due to their inhibitory effects, these dyes must be introduced post-reaction, increasing contamination risks. Colorimetric measurements are among the most straightforward of all of the detection methods. Several dyes have been reported to be useful, such as hydroxynaphthol blue (HNB) [30] and malachite green (MG) [31]. HNB requires the operator to distinguish between blue and violet, which can be ambiguous. In contrast, MG only requires distinction between blue and transparency.

LAMP has also been applied for the detection of *L. infantum*, both in dogs and humans [32, 33]. Pan-leishmania LAMP is also reported by Karani et al.*,* Mikita et al. and Nzelu et al. [26, 31, 34]. However, *Leishmania*–specific colorimetric LAMP from clinical samples has never been described.Therefore, in this study, we developed a LAMP method using MG to detect *Leishmania* DNA from crude clinical samples. These data can be useful for the deployment of LAMP in field settings and can further enable detailed surveying of *L. siamensis* and *L. martiniquensis* in Thailand.

## Methods

### Primer design

**Fig. 1:**
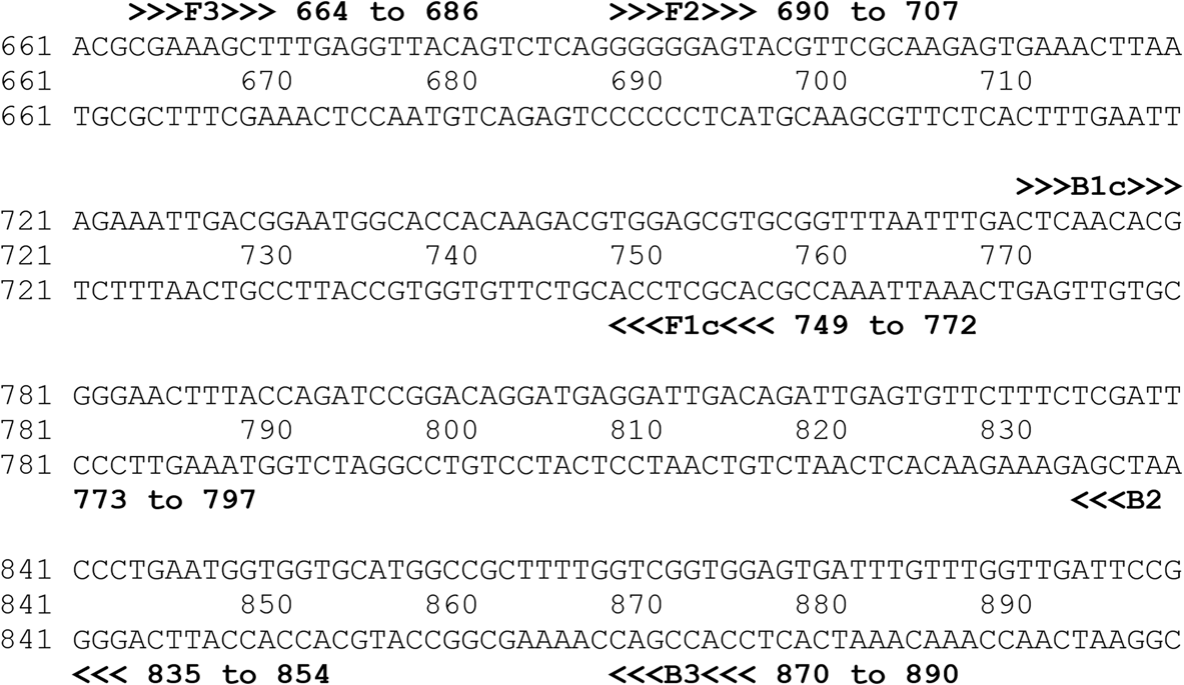
Targeted Amplification Region on the 18S Ribosomal RNA Gene of *L. martiniquensis* (KJ467218.1)

To develop a pan-leishmania assay, we chose the highly conserved 18S ribosomal RNA gene, as in previous pan-leishmania assays [26, 31, 34]. A consensus sequence was made from nine different *Leishmania* species, including *L. tropica* [GenBank: KF041809.1], *L. martiniquensis* [KJ467218.1], *L. mexicana* [KF041806.1], *L. hertigi* [KF041804.1], *L. donovani* [KF041801.1], *L. chagasi* (syn. *L. infantum*) [KF041797.1], *L. infantum* [KF302752.1], *L. amazonensis* [KF302746.1], and *L. enriettii* [KF041798.1]. However, *L. siamensis* was not included in this process due to the absence of its sequences on GenBank. The consensus sequence was imported into the PrimerExplorer version 4 software (http://primerexplorer.jp/elamp4.0.0/index.html), and primers were designed to avoid any mutations that were presented. To ensure optimality, primers with the lowest change in Gibb’s free energy (∆G) for dimer formation and the highest change in hybridization ∆G were chosen. The primers’ thermodynamic properties were further validated using the OligoCalc software (http://www.basic.northwestern.edu/biotools/oligocalc.html). A final verification of specificity was performed using Basic Local Alignment Search Tool (BLAST) (http://blast.ncbi.nlm.nih.gov/Blast.cgi) analysis against human DNA and any other organisms included in the differential diagnosis of leishmaniasis. The resulting primers are shown in Table 1 and Fig. 1, and were synthesized by BioDesign Co., Ltd, Pathumthani, Thailand.

**Table 1:** Primer sequences used in the study

Primer	Sequence (5’→ 3’)
FIP (F1c-F2)	GTCAAATTAAACCGCACGCTCCACGGGGGAGTACGTTCGCAA
BIP (B1c-B2)	TCAACACGGGGAACTTTACCAGATCACCACCATTCAGGGAATCGA
F3	CGAAAGCTTTGAGGTTACAGTCT
B3	CAAACAAATCACTCCACCGAC

### Quantitative polymerase chain reaction

The 25 μl reaction mixture contained 12.5 μl of 2× reaction mix from the SuperScript® III Platinum® SYBR® Green One-Step qRT-PCR Kit (Life Technologies, USA), 0.08 μM of F3 primer, 0.08 μM of B3 primers, and 1 U of BIOTAQ™ DNA Polymerase (Bioline, Germany). The reaction was performed using the CFX96™ real-time PCR system (Bio-Rad Laboratories, USA) and was programmed as followed: 3 min of initial denaturation at 95 °C, 40 cycles of denaturation at 95 °C for 20 sec, annealing at 50 °C for 30 sec, extension at 72 °C for 40 sec, and fluorescence data acquisition at 77 °C. Upon completion, a final extension at 72 °C for 10 min ensued. The C_q_ (quantification cycle) was defined to be 10 times the standard deviation of the baseline. A standard curve was generated using DNA dilutions in triplicates, and the efficiency was determined to be 98.44 % with a linear range spanning 6 orders of magnitudes. Specificities of all samples were verified using melting curve analyses, which demonstrated a single peak at 83.5 °C.

### Loop-mediated isothermal amplification

The LAMP reaction mixtures (25 μl) were based on that described by Tomita et al. [28], which contained 1× Isothermal Amplification Buffer (New England Biolabs, USA), 8 mM MgSO_4_, 0.8 M Betaine (Sigma-Aldrich, USA), 1.4 mM each of dATP, dCTP, dGTP, and dTTP (SibEnzyme, Russia), 40 pmol of FIP primer, 40 pmol of BIP primer, 10 pmol of F3 primer, 10 pmol of B3 primer, and 8 U of *Bst* 2.0 WarmStart® DNA Polymerase (New England Biolabs, USA). In addition, the colorimetric indicator, 0.008 % MG (Sigma-Aldrich, USA) was added. The reaction was performed at 65 °C for 75 min using the Veriti® 96-well Thermal Cycler (Life Technologies, USA). Visualization of the LAMP products was performed using 2.5 % agarose gel electrophoresis at 10 V/cm in 1× TAE buffer.

### Promastigote culture

*L. siamensis* isolate PCM2 and *L. martiniquensis* isolate CU1 were used in this study and were derived from bone marrow aspirates of infected patients. The culture media was Schneider’s insect medium (Sigma-Aldrich, USA) supplemented with 10 % fetal bovine serum, 100 U of penicillin (Sigma-Aldrich, USA), and 100 μg/ml streptomycin (Sigma-Aldrich, USA). The cultures were incubated at 25 ± 2 °C, and subculturing was done every 2–3 days at a ratio of 1:2.

### Reference DNA

All *Leishmania* DNA samples used in this study were extracted using the Invisorb® Spin Tissue Mini Kit (Stratec Biomedical AG, Germany). While *L. siamensis* and *L. martiniquensis* DNA were derived from cell culturing, *L. aethiopica*, *L. braziliensis*, *L. donovani*, and *L. tropica* DNA were derived from tissue biopsies of patients with imported leishmaniasis. *Trypanosoma brucei* DNA was extracted from a permanent slide sample, while *Trichomonas vaginalis* and *Giardia lamblia* DNA were isolated from infected patient samples at King Chulalongkorn Memorial Hospital. All DNA samples were of sufficient quality, as indicated by their optimal 260/280 and 260/230 ratios.

### Detection limit

To generate standard parasite concentrations and assess LAMP’s tolerance to inhibitors that can be presented in the saliva, 10-fold dilutions of *L. siamensis* from 10^7^–10^0^ parasites/ml were made using either 1× phosphate-buffered saline (PBS) or human saliva. Each dilution was divided into two portions. One portion was extracted for DNA using the Invisorb® Spin Tissue Mini Kit (Stratec Biomedical AG, Germany), and the other was boiled at 100 °C for 10 min, as described previously [26].

To simulate infected blood, 100 μl dilutions of *L. siamensis* in human blood were made as described above and divided into two portions. The first portion was added with Triton X-100 to a final concentration of 1 %, and boiled at 100 °C for 10 min, which resulted in a coagulum. Next, 50 μl of ddH_2_O was added, and the coagulum was broken up by vigorous agitation with a pipette tip. The second portion was subjected to DNA extraction using the Invisorb® Spin Blood DNA Mini Kit (Stratec Biomedical AG, Germany); 2.5 μl of each resultant was directly introduced to both qPCR and LAMP.

### Clinical samples

Peripheral blood and saliva were obtained from two patients.

Patient 1 was initially reported by Phumee et al. [35]. In short, the patient was a 49-year-old man who was HIV positive and presented with multiple nodules on his body. Microscopy and cell culturing revealed the presence of *Leishmania* parasites in the nodules, and molecular analysis confirmed *L. martiniquensis* infection [16]. He was successfully treated with amphotericin B and itraconazole. Blood, saliva, and tissue biopsy were obtained. The blood was treated in the same manner as described above, while the saliva and tissue biopsy (drenched in 1× PBS) were boiled at 100 °C for 10 min. 2.5 μl of the supernatant were used as the template.

Patient 2 was described by Chusri et al. [10]. He was a 30-year-old man who was HIV positive. Similar to Patient 1, he had multiple papules on his skin, but this patient also had internal organ involvements. *Leishmania* parasites were microscopically confirmed to be infiltrating the bone marrow and ulcers. Further molecular data confirmed this *Leishmania* species to be *L. martiniquensis* [16]. He was also successfully treated with amphotericin B and itraconazole. Blood, saliva, and bone marrow biopsy were obtained. The saliva and blood was treated as described earlier, and the bone marrow biopsy was treated in the same manner as the blood.

## Ethical statement

This study was approved by the Institutional Review Board on Human Research of the Faculty of Medicine, Chulalongkorn University (COA No. 725/2013).

## Results

**Fig. 2:**
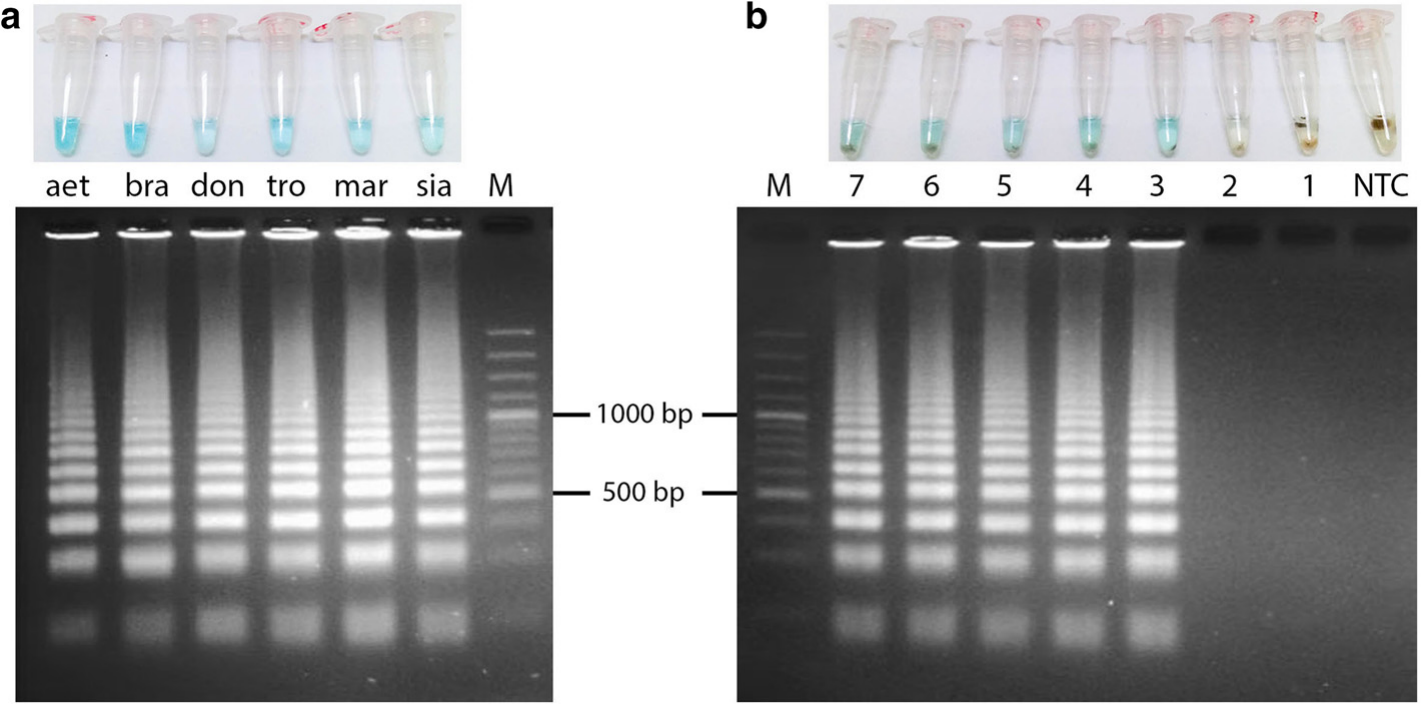
Detection of LAMP Products by MG-based Colorimetric Changes and Gel Electrophoresis. **a** LAMP was able to detect multiple species of *Leishmania*. (aet = *L. aethiopica*; bra = *L. braziliensis*; don = *L. donovani*; tro = *L. tropica*; mar = *L. martiniquensis*; sia = *L.siamensis*) **b** LAMP could detect *L. siamensis* in the presence of whole blood. Ten-fold dilutions of *L. siamensis* in whole blood (log parasites/ml) were lysed, boiled, and subjected to LAMP. The black precipitates are coagulated blood

Both qPCR and LAMP successfully amplified regions that were specified by the newly designed primers. For LAMP, successful amplification was associated with MG’s characteristic light blue color, whereas failed amplification was associated with transparency (Fig. 2). Gel electrophoresis of the LAMP products also exhibited their characteristic “mixture of stem–loop DNAs with various stem lengths, and cauliflower-like structures with multiple loops” [25], which further confirmed their successful amplifications.

The detection limits (log_10_ parasites/ml) of *L. siamensis* for LAMP, as defined by the appearance of its light blue color, and qPCR, as defined by fluorescence above the C_t_, are shown in Table 2.

**Table 2:** Detection limits of LAMP and qPCR under various conditions (log parasites/ml) (F* = Fail to amplify)

Diluent	Method	LAMP	qPCR
1X PBS	Boiled	3	5
	Extracted	3	4
Saliva	Boiled	3	4
	Extracted	4	4
Whole blood	Boiled	3	F*
	Extracted	4	5

For qPCR, our results indicated that the direct use of the samples was possible but was associated with increases in the detection limits. However, whole blood could not be used, due to the total inhibition of *Taq* polymerase.

In the case of LAMP, which is more tolerant of PCR inhibitors, removal of the extraction process allowed a lower detection limit of 10^3^ parasites/ml across all samples. Crude samples did not affect the properties of MG as all of the positive samples displayed MG’s characteristic color. The use of whole blood did shift the color toward a greenish tone, whereas the negative samples were yellow in color (Fig. 2). However, an excessive amount of whole blood or the presence of uncoagulated blood could prevent the discrimination between positive and negative results, as hemoglobin absorption spectrum overlapped that of MG.

**Fig. 3:**
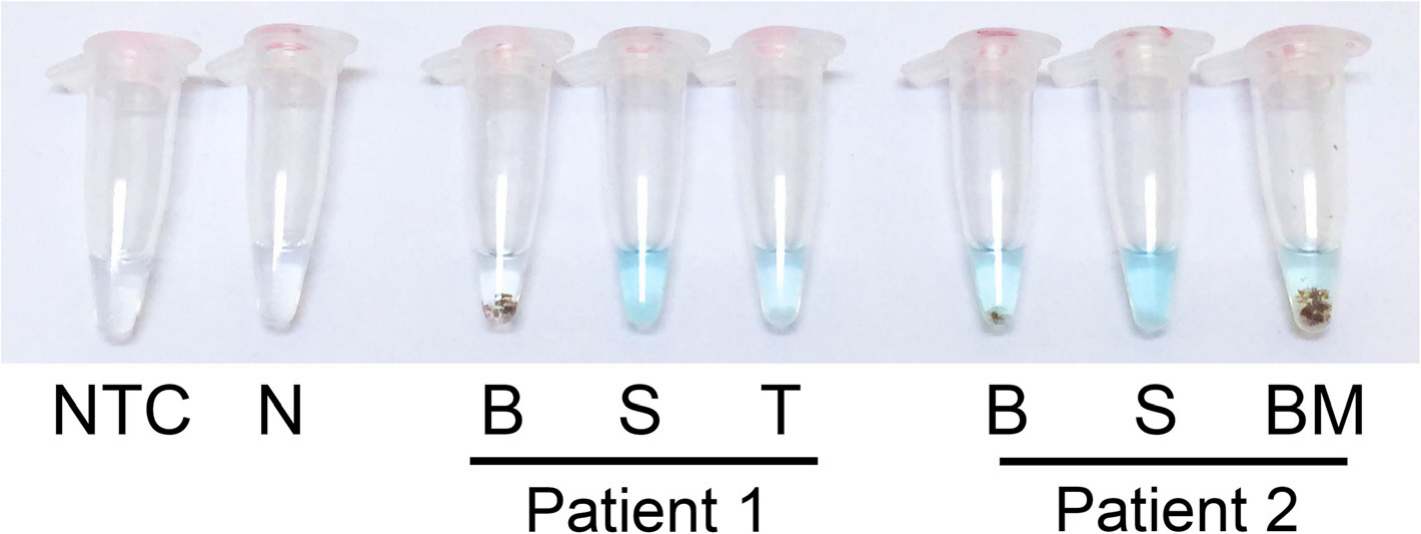
LAMP Could Be Used Directly with Clinical Samples. Crude clinical samples were directly introduced into the LAMP reaction after being subjected to heating. (NTC = no template control; N = healthy patient control; B = blood; S = saliva; T = tissue biopsy; BM = bone marrow)

Clinical samples also yielded useful information in microscopically diagnosed patients. Patient 1, who had CL, had detectable *Leishmania* DNA in his saliva and tissue biopsy. Patient 2, in contrast, who had CL and VL, had the DNA presented in his bone marrow, blood, and saliva (Fig. 3).

## Discussion

With an increasing number of cases, leishmaniasis is now an emerging infectious disease in Thailand. However, current diagnostic methods require experienced personnel, advanced facilities, and a large amount of time. Thus, simplification is now critical to bring diagnostics to point-of-care settings. Therefore, we developed the LAMP method to complement leishmaniasis diagnostic process and to facilitate epidemiological studies of leishmaniasis in Thailand.

Molecular techniques have exploited multiple genes to detect *Leishmania*, most of which are high-copy-number genes, including cysteine protease B [36], *gp63* [37], internal transcribed spacer 1 (ITS1) [38], 18S ribosomal RNA [26, 31], and minicircle kinetoplast DNA [24]. As expected, comparative studies have shown that the minicircles, which have the highest copy number, yield the highest sensitivity [39, 40]. However, minicircles are highly variable and due to LAMP’s requirement of six conserved regions, it is not feasible to design pan-leishmania LAMP primers that target minicircles. In our approach, we chose the 18S ribosomal RNA gene, which represents a balanced trade-off between copy number and variability. As Karani et al.*,* Mikita et al. and Nzelu et al. recently reported, LAMP has been shown to detect most species of *Leishmania* [26, 31, 34]. Here, we also experimentally confirmed the detection of *L. martiniquensis* and *L. siamensis*. Our detection limit was comparable to that of Mikita et al. (10^3^ parasites/ml), but was 10-fold higher than that of Nzelu et al. (10^2^ parasites/ml) [26, 31].

We detected *Leishmania* DNA in the blood and bone marrow, and in the tissue and saliva of the VL and CL patient, respectively. Our findings agreed with others that *Leishmania* DNA can be found in multiple noninvasive sources, including saliva (CL, VL) [10, 12, 41], skin swabs (CL) [26] and peripheral blood (VL) [42, 43]. We recommend the use of multiple DNA sources to reduce the probability of false negatives.

Our results also suggested that LAMP could be used to detect *Leishmania* DNA from crude clinical samples without compromising the detection limit. Furthermore, the use of crude samples even lowered the detection limit by 10-fold (10^3^ parasites/ml), but the same could not be said with qPCR, which increased this limit by 10-fold. We suspected that in the case of LAMP, the removal of inhibitors did not compensate for the loss of DNA from the extraction process, as in the case of qPCR. These results are in concordance with other studies that used boiled samples in LAMP [26, 44, 45]. In addition, of all of the DNA preparation methods compared, Sun et al. [46] reported that simple boiling results in the highest sensitivity.

Furthermore, the use of MG greatly facilitates the interpretation of results as it is highly discernable and consistent [31]. Because MG is inexpensive and can be stored at room temperature, this dye could tremendously increase the applicability of LAMP in the field.

Earlier attempts to couple direct blood samples with a colorimetric detection method were unsuccessful, due to the intense color of hemoglobin. To solve this problem, we induced the precipitation of the blood using its own coagulation system. We initially lysed the blood and its accompanying parasites with Triton X-100 and then promoted coagulation by boiling the sample. After boiling the sample, the coagulum was pulverized using a pipette tip. From this approach, we could clearly distinguish the results using MG, even with 2.5 μl of whole blood.

Because LAMP can be performed isothermally using a simple heat block, stable electricity is not required. Recently, using phase-change material, LaBarre et al. [47] developed a stable heat block that did not require electricity. Microfluidics lab-on-a-chip for LAMP has also been developed and has enabled highly multiplexed reactions and further simplification [48]. Moreover, Tanner et al. [49] reported that the LAMP reaction mixture can be left at 37 °C for two hours without compromising the detection limit. LAMP mixtures have also been lyophilized with a reported storage time at room temperature of at least seven months [50].

Nevertheless, because of the low prevalence of leishmaniasis in Thailand, we could only obtain a small number of microscopically diagnosed patients. Future work in a statistically significant group of patients is required to warrant the performance of this method.

Also, LAMP is particularly prone to contamination due to the large amount of DNA that it can generate, and its capability to amplify minute amounts of DNA. During this study, we nevertheless encountered multiple contamination issues. Therefore, we recommend that post-amplification reaction mixtures should not be opened due to aerosolization risks, and that all proper precautions be taken [51]. To prevent false positive results, no template control and healthy patient control should also be used. We also tested our assay specificity with *T. vaginalis* and *G. lamblia* DNA, with satisfying results. However, our assay did cross-react with DNA from *Trypanosoma* sp., a closely related protozoon (data not shown). Nevertheless, as each protozoon has a distinct set of clinical presentations, this cross-reaction would not impose a significant risk of misdiagnosis.

## Conclusion

Sensitive molecular techniques to diagnose leishmaniasis have been introduced; nevertheless, due to their complexities, they have not yet been in widespread use. However, as our results demonstrated, LAMP could be used with unpurified clinical samples without compromising sensitivity and specificity, and its results could be unambiguously interpreted using MG.

With the advent of LAMP, molecular techniques can now be seamlessly integrated with field diagnostics. We anticipate that this combination will be crucial for surveying and controlling leishmaniasis in Thailand. However, further evaluation with a large cohort of patients will be required before the assay can be confidently deployed.

## Abbreviations

CL: Cutaneous leishmaniasis
DAT: Direct agglutination test
ELISA: Enzyme-linked immunosorbent assay
ICT: Immunochromatographic test
IFA: Indirect fluorescence antibody
LAMP: Loop-mediated isothermal amplification
MCL: Mucocutaneous leishmaniasis
MG: Malachite green
qPCR: Quantitative polymerase chain reaction
VL: Visceral leishmaniasis
